# Traditional Glue, Adhesive and Poison Used for Composite Weapons by Ju/’hoan San in Nyae Nyae, Namibia. Implications for the Evolution of Hunting Equipment in Prehistory

**DOI:** 10.1371/journal.pone.0140269

**Published:** 2015-10-28

**Authors:** Lyn Wadley, Gary Trower, Lucinda Backwell, Francesco d’Errico

**Affiliations:** 1 Evolutionary Studies Institute and Centre of Excellence in Palaeosciences, University of the Witwatersrand, Private Bag 3, WITS 2050, South Africa; 2 School of Geography and Archaeology, University of the Witwatersrand, Private Bag 3, WITS 2050, South Africa; 3 Université de Bordeaux 1, UMR 5199 PACEA, Equipe Préhistoire, Paléoenvironnement, Patrimoine, 33405 Talence cedex, France; 4 School of Geosciences, University of the Witwatersrand, Private Bag 3, WITS 2050, South Africa; University of Oxford, UNITED KINGDOM

## Abstract

Ju/’hoan hunters from Nyae Nyae, near Tsumkwe in Namibia, demonstrate the manufacture of three fixative pastes made from plant extracts, and poison made from grubs and plant extracts. *Ammocharis coranica* and *Terminalia sericea* produce simple glue. *Ozoroa schinzii* latex mixed with carbonized *Aristeda adscensionis* grass is a compound adhesive. Composite poison is made from Chrysomelid grub viscera mixed with salivary extracts of *Acacia mellifera* inner bark and the tuber sap of *Asparagus exuvialis*. In order to document potential variability in the *chaîne opératoire*, and to eliminate inherent biases associated with unique observations, we studied manufacturing processes in three separate Nyae Nyae villages. Although there are methodological similarities in the Nyae Nyae area, we observed a few differences in contemporary traditions of poison manufacture. For example, some hunters make powder from *Asparagus exuvialis* tuber sap by boiling, reducing, hardening and grinding it, while others simply use heated sap. The Ju/’hoan hunting kit provides insights for archaeologists, but we must exercise caution when looking for continuity between prehistoric and historical technical systems. Some traditions have been lost to modern hunters, while others are new. We should also expect variability in the Stone Age because of geographically restricted resources. Simple glue, compound adhesive, and poison recipes identified in the Stone Age have no modern equivalents. By about 60,000 years ago at Diepkloof, simple glue was used for hafting tools, but at similarly-aged Sibudu there are recipes that combine red ochre powder with plant and/or animal ingredients. At Border Cave, novel poisons and compound adhesives were used in the Early Later Stone Age. It is possible that the complexity that we record in the manufacture of fixative pastes and poison used by Ju/’hoan hunters represents a hafting system both similar to and different from that observed at the Stone Age sites of Diepkloof, Sibudu, and Border Cave.

## Introduction

Composite weaponry seems to have originated in Europe and Asia in the Middle Palaeolithic (MP) and in Africa during the Middle Stone Age (MSA). Hafting of tools was an integral part of MP and MSA technology. Western European Neanderthals heated birch bark for glue by Marine Isotope Stage 6 (MIS 6, that is, ~200,000–130,000 years ago) at the Campitello quarry site in Central Italy [1,2]. Stone tools from an occupation ~120,000 (120 ka) years old, in Inden-Altdorf, Germany, were hafted with birch bark pitch [3] and it was also discovered at Königsaue, Germany, where the geological-stratigraphic context suggests ages older than 80 ka [4]. Bitumen was used for hafting ~33–29 ka ago at Gura Cheii-Râsnov Cave, Romania [5]. In Asia, three bitumen-bearing Mousterian artefacts were found in Hummal (Central Syria) [6] and at Umm el Tlel, bitumen was used for hafting from at least 70 ka onwards [7–12] (Table 1).

**Table 1:** Residues that imply hafting on archaeologically-recovered stone tools.

Site	Residue	Association	Reference
Enkapune Ya Muto, Kenya	Ochre stains*	Early LSA, >40 ka	[22]
Diepkloof Rock Shelter, South Africa	*Podocarpus* resin, mixed with bone fragments and quartz grains *	MSA, MIS 5d—MIS 3	[16]
Rose Cottage Cave, South Africa	Plant exudates, ochre stains	MSA, 68–60 ka	[23,60,61]
Die Kelders, South Africa	Ochre stains	MSA	[62]
Border Cave, South Africa	Lump of beeswax, *Euphorbia tirucalli* resin and possibly egg *	~ 44 ka	[15]
Border Cave, South Africa	resin from *Podocarpus* bark *	MSA and LSA	[18]
Sibudu Cave, South Africa	Ochre, resin/gum, fat or wax; unidentified brown stains	MSA, 71–38 ka	[20, 24–26, 63–66]
Sibudu Cave, South Africa	ochre, coniferous resin *	MSA, 65 ka	[17]
Apollo 11, Namibia	unidentified mastic	MSA, 27–25 ka	[19]
El Kowm basin (Umm El Tlel & Hummal), Syria	Bitumen *	MP, ~71 ka; ~40 ka	[7,8,10,11,67]
Hummal, Syria	Bitumen *	MP	[68]
Umm El Tlel, Syria	Bitumen	MP, ~70 ka	[12]
Quneitra, Israel	Unknown material, degraded	MP, 54 ka	[69]
Hummal spring, Syria	Bitumen *	MP	[6]
Les Vachons, France	Birch-bark tar *	Upper Paleolithic	[70]
Königsaue, Germany, lignite mining pit	Birch-bark tar *	~ 43.8 ka, ~ 48.4 ka	[71]
Königsaue, Germany, lignite mining pit	Birch-bark tar *	> 80 ka	[4]
Campitello quarry, Italy	Birch-bark tar *	late Middle Pleistocene, MIS 6	[1]
Inden-Altdorf, Germany	Birch-bark tar *	~120 ka	[3]
Gura Cheii-Râşnov Cave, Romania	Bitumen *	MP and Upper Paleolithic	[5]

* denotes chemical identification.

The use of multi-component spears may be as old as 500 ka ago in both East [13] and South Africa [14] and, if this is the case, hafting has great antiquity. Stone points designed for use as spearheads do not necessarily require glue; they can be fastened to shafts with sinew or plant twine, but the addition of fixative paste would have produced a more robust and reliable attachment. At present there is no evidence for the use of fixative pastes early on in the African MSA because glues and adhesives only remain as residues on stone tools if there is good organic preservation in the archaeological site concerned. The oldest plant resins chemically identified on MSA stone tools come from Diepkloof, Sibudu and Border Cave in South Africa [15–17] (Table 1). Using GC-MS analysis, *Podocarpus* sp. resin was securely identified on a MSA tool with an age of ~56 ka from Diepkloof [16], on stone tools from Border Cave ~43−42.5 ka ago [18]. Two Sibudu tools, from layers with ages of ~65 ka and ~62 ka, retain traces of resin identified chemically as conifer, most likely from *Podocarpus* [17]. The resin was mixed with several ingredients including red ochre and animal fat (Villa et al., 2015). A flake in a 49 ka layer of Sibudu also yielded a milk and ochre blend interpreted as paint [17], so future chemical analyses of African tool residues are likely to reveal further novel and unexpected technologies. A mixture of beeswax, toxic *Euphorbi*a sp. resin, and possibly egg, dated to ∼40 ka, may have been a hafting recipe at Border Cave, and a poison applicator stick (~24 ka) revealed traces of poison [15].

Chemical analyses have not yet been conducted widely on African stone tools; visual inspection and microscopy inform most observations. Single component mastic was part of hafting materials at Apollo 11 [19], but compound adhesives with mixtures of ochre, plant gum and fat or wax were commonly identified at Sibudu [20,21]. Sometimes a mineral component of adhesives retains a coloured trace on stone tools, either with or without accompanying organic residues. Ochre, used as a loading agent in some adhesives, remains as a red trace on stone tools from, for example, Enkapune ya Muto in East Africa [22], Rose Cottage Cave in Free State, South Africa [23] and Sibudu [20,21, 24–26] (Table 1). Red ochre is particularly prevalent on the backed (blunted) edges of backed tools that were presumably to facilitate hafting. However, at Sibudu, not all backed tools have red ochre adhesive traces; some have black adhesive traces, while others have plant gum or resin glue that can be visually detected through microscopy [26,27]. Small quartz backed tools from Sibudu appear to have fixative paste that generally lacks ochre [28], thus, different recipes for glues and adhesives may have been used on stone tools in archaeological sites. The use of *Podocarpus* resin, and the absence at Diepkloof of ochre-loaded adhesives, was interpreted as a regional hafting tradition [16]. Some differences in the use of fixative pastes may be the result of local conditions, but other factors may be at play. A variety of paste types and hafting angles was used at Sibudu, and this diversity may have arisen because a toolkit with dissimilar composite tools is represented. Certainly microscopic analysis of the stone tools suggests that this may be the case and that particular fixatives are associated with specific tool uses. At 44 ka ago in Border Cave there are small bone points with possible marks of ownership coloured with red ochre, and, together with the poison applicator, these artefacts imply the use of hunting devices similar to those used by San hunters historically [15].

The archaeological evidence for hafting techniques appears to confirm the sorts of regional and possibly time-related traditions that are also implied by bone [29] and stone technology [30,31]. However, the archaeological evidence is mute on the behavioural, technological and cognitive complexity involved in the production and use of fixative pastes for hunting and other types of equipment. Using an ethno-archaeological approach seems a useful way to evaluate the archaeological innovations at a broader scale and to compare, where possible, archaeological data with the present day hunter-gatherer equipment and technology. Available ethnographic accounts of San hafting practices (for example, [32–35] do not provide the necessary details of the *chaîne opératoire* to enable us to follow the collection of raw materials, to learn about their properties, then to observe the precise sequences of actions, and discover their functions. Archaeologists need that detail in order to establish meaningful comparisons between ethnographic and prehistoric artefacts. We therefore studied three independent villages in the Nyae Nyae Conservancy to document variability and similarities in the hafting *chaîne opératoire*, and to eliminate biases inherent in unique observations. We aimed to explore the degree of elasticity of the *chaîne opératoire* involved in the hafting process, for example, which elements remain constant, or which may change as a consequence of variation between individuals or groups, in time and across space. We decided to examine poison recipes in addition to glues and adhesives because poisons and fixative pastes are frequently present on the same modern hunting weapon. A similar situation may be represented at Border Cave, where the ~40 ka old beeswax and *Euphorbia* mixture represents a poisonous adhesive [15].

We distinguish two types of fixative paste here: first, *glue* which is a single component product requiring little preparation other than, perhaps, heating or mixing with water or saliva. Plant glue may originate either from gum, a saccharide that is soluble in water, or from resin which is a volatile, fluid terpene, insoluble in water. Secondly, *adhesive* is a compound product irreversibly altered through the combination of disparate ingredients and sometimes also through heating. The creation of adhesive is a multifaceted process that involves carefully planned thought and action sequences. It has been argued elsewhere [21,36] that the presence of adhesive in the archaeological record is a proxy for complex cognition.

## Background to the Ju/’hoan San of Nyae Nyae

**Fig 1:**
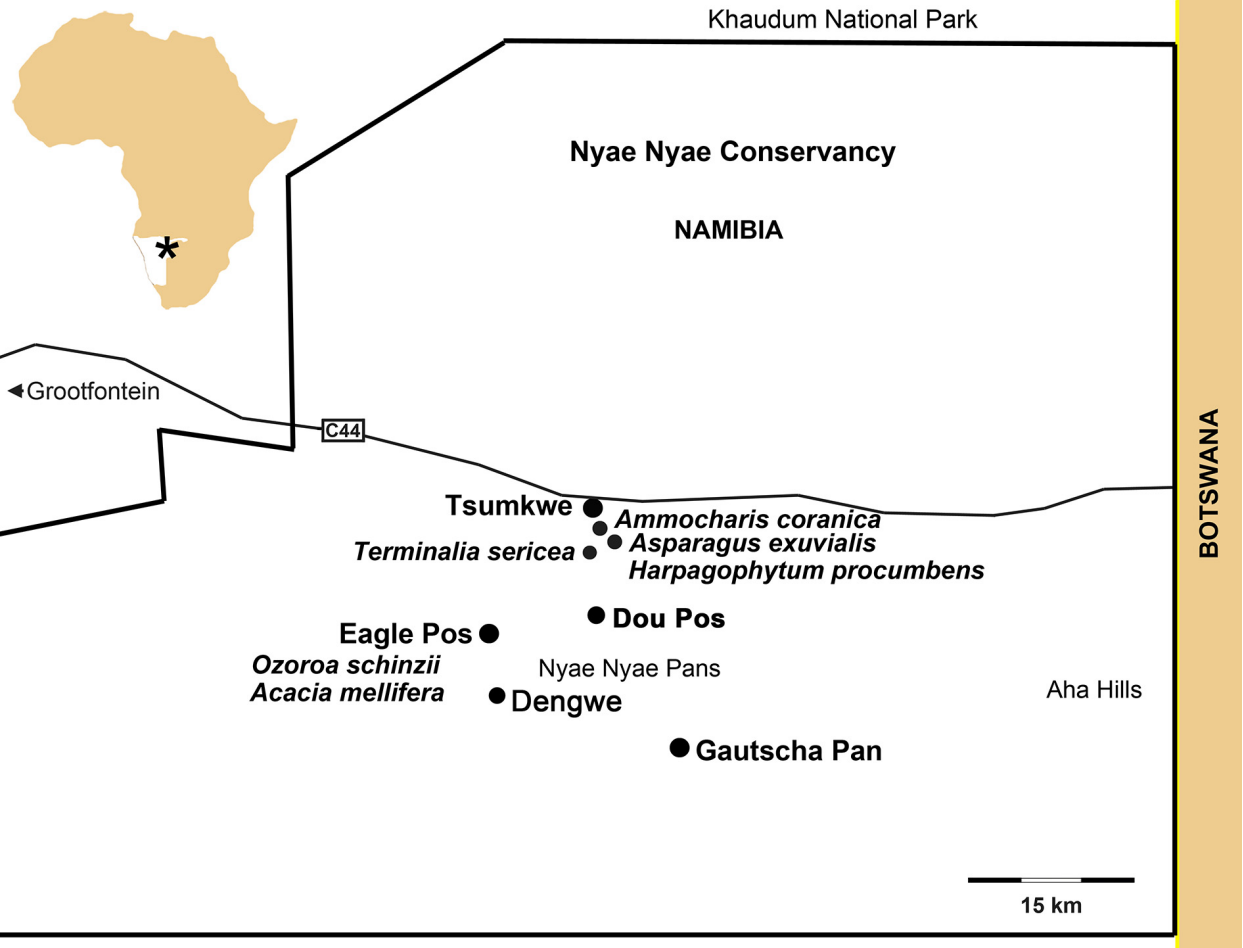
Map of Nyae Nyae Conservancy showing the localities discussed in the text. The plant names with white circles indicate the collection points for the material used at Eagle Pos.

The Nyae Nyae region is named for the pan about 50 km from the Namibia/Botswana border. Several pans and waterholes fill after the summer rains in this open savanna region. In the 1950s when the Marshall family lived and worked with Ju/’hoan communities at Tsumkwe and in the Nyae Nyae area, they were still independent hunter-gatherers even though they were not unaffected by herding and farming communities [32–34]. The communal Nyae Nyae Conservancy (Fig 1) was established in 1998. Covering 8,992 square kilometres, it is home to approximately 2,300 Ju/’hoansi (pronounced jewtwasi) people (note that we follow Biesele and Hitchcock [37] in using Ju/’hoan as the adjectival form), and some Herero, who endlessly push for land to graze their large herds of cattle. To some extent, Ju/’hoansi of the area maintain traditional hunting and gathering practices, but their hunting activities are severely limited by government quotas [37], which result in hunters forfeiting hunting in favour of cash, which is paid to the Community Trust by trophy hunters. Furthermore, important traditional plant gathering has been kerbed by the demarcation of the nature reserve which denies them access to both mongongo nut tree groves and tsin bean patches [37,38]. The sale of crafts to cooperatives and directly to tourists provides a little cash. Some people have taken up herding of livestock due to the importance of cattle wealth in the region, but the people we worked with spoke derisively of “Herero buffalo” (cattle).

Ju/’hoansi speak a! Kung dialect, indeed, they were referred to as! Kung people in the publications of the 1950s to 1970s (for example, [32–34]). Our contact and interpreter was ≠oma Tsamkxao (Leon), son of Tsamkxao ≠oma (father) and //uce N≠amce/ui (mother), and grandson of ≠oma Tsamkxao and! U. ≠oma Tsamkxao, known by Marshall [33] and Marshall Thomas [32] as ≠Toma, the Leader from /Gam. He is deceased, but his son, Tsamkxao ≠oma (Chief Bobo), almost 80 years old, is considered “chief” of the Ju/’hoansi, and he lives with his wife in Tsumkwe. Our interpreter, ≠oma Tsamkxao (Leon), works as a guide at the Tsumkwe Country Lodge. He helped John Marshall to complete his last documentary, *A Kalahari Family*, in 2003. Leon and the Nyae Nyae Conservancy recruited, on our behalf, six experienced hunters. Two of them, /uce N≠amce and ≠oma /Kunta, came from Eagle Pos (a village west of Tsumkwe, S 19. 42. 41.4; E 20. 24. 03.6). Another two were from the village of Dengwe (Den/ui), and the last two from the village of Dou Pos. The hunters were employed to take us on collecting expeditions that would be followed by demonstrations of glue, adhesive and poison manufacture. LW and GT worked with ≠oma Tsamkxao (Leon), /uce N≠amce and ≠oma /Kunta in March 2015 and observations were recorded with notebooks, digital cameras, a Sony Handycam video camera and two Chesty Go-Pro video cameras. The same method was employed by GT and LB who worked with /ui G/’aqo and G≠xao Kgao from Dengwe and //ao ≠Oma and /Kunta Boo from Dou Pos in September 2014, and FD and LB who worked with //ao ≠Oma from Dou Pos in May 2013 (without Go-Pro cameras).

The Nyae Nyae Conservancy management committee, together with Tsamkxao ≠oma (Chief Bobo), approved our research permit to work with hunters in the area and the committee assisted with the selection of hunters to participate in the project. The University of the Witwatersrand Ethics Committee approved this study and provided advice on creating the consent forms to be signed by the hunters. The participants have provided consent for publication; the individuals in this manuscript have given written informed consent (as outlined in PLOS consent form) to publish these case details. The consent forms state that the hunters will demonstrate the collection of ingredients for making glues, poison and weapons and that they will be photographed and filmed while doing these tasks. The form makes it clear that they have the right not to answer questions and to request that some photographs are not made public. The Namibian Ministry of Environment and Tourism issued a Research/Collecting Permit, permit number 2029/2015. The South African Department of Agriculture, Forestry and Fisheries issued a Veterinary Import Permit for crafts, permit number 13/1/1/8/2/0-201502000094. We purchased several hunting kits for study in the laboratory; such kits are readily available for sale from co-operatives in Tsumkwe.

**Fig 2:**
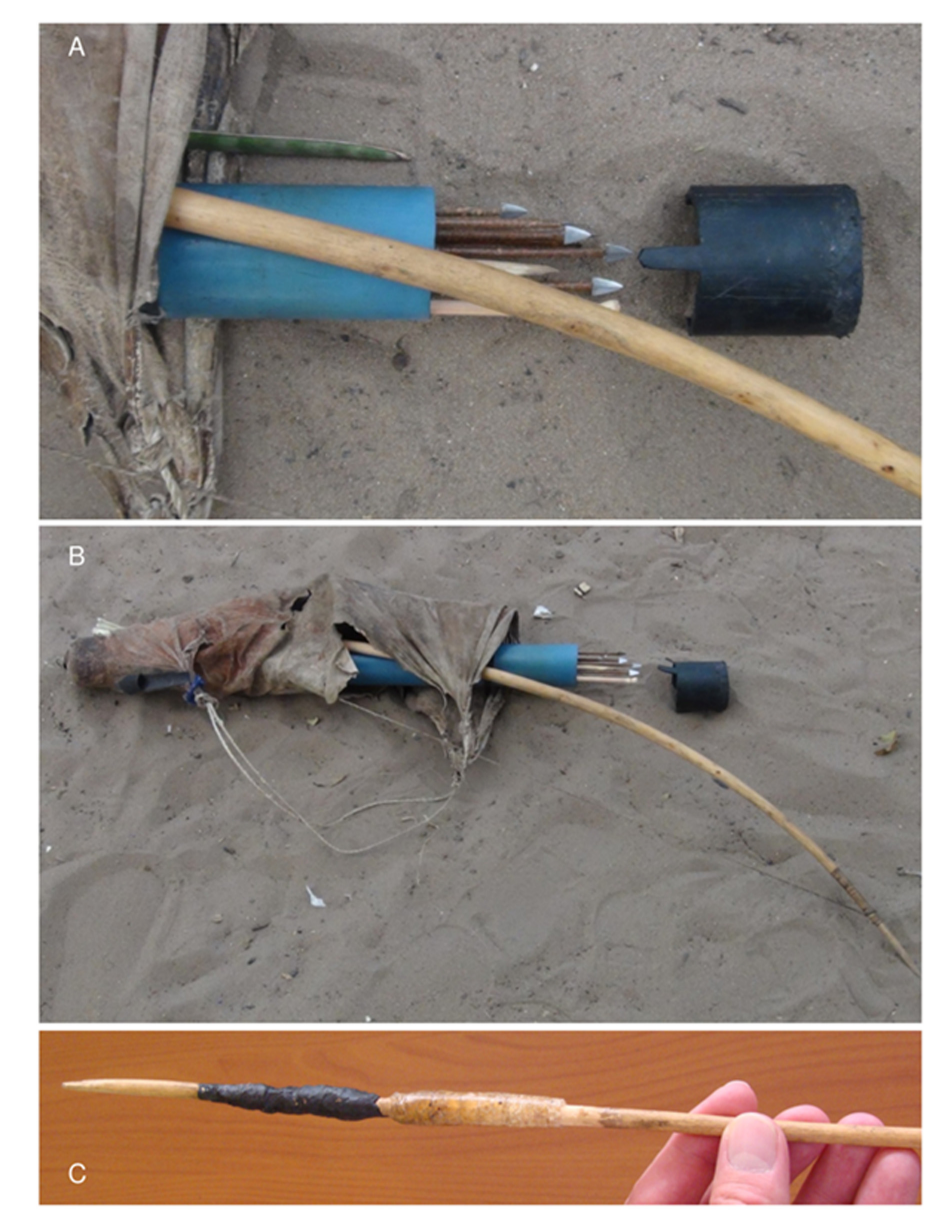
Hunting equipment from Dengwe and Dou Pos. A. Detail of Dengwe quiver with arrows. Note the *Sansevieria* leaf above the quiver; B. Dengwe hunting bag with bow and quiver (made from blue plastic pipe); C. Black *Ozoroa schinzii* adhesive and yellow *Terminalia sericea* gum on *Grewia flava* stick that is both an applicator and storage device (from Dou Pos).

The traditional Ju/’hoan hunting kit comprises a leather hunting bag that contains bow and quiver, spear, and wooden digging stick [34]. Traditional quivers are made from a sheath of root bark of either *Acacia erioloba* or *A*. *luederitzii*, but many hunters nowadays utilize lengths of plastic piping for quivers (Fig 2). In each hunter’s quiver, together with a supply of ready arrows and arrow components, is a male and female fire stick, and a stick that houses glue and has a bevelled end to act as a spatula for poison applications (Fig 2C). Sometimes the quiver holds a reserve of Chrysomelid grubs, the primary ingredient of arrow poison. The arrows are metal-tipped, made from beaten fencing wire (Fig 2). Their manufacture will be described later. Sometimes bone arrows are incorporated into the hunting gear. The glue on the applicator stick comprises two products, a hard, clear, honey-coloured plant gum that is simple glue, and a pliable, black mixture that is an adhesive (Fig 2C). The glue stick pastes are used during the manufacture or renovation of arrows. The black adhesive is made from *Ozoroa shinzii* (Engler) (resin bush) latex mixed with powdered, carbonized *Aristeda adscensionis* grass, whereas the clear glue is gum from *Terminalia sericea* (Burch.) (silver cluster-leaf). A hard ball of dark brown paste may also be carried in the quiver; this is *Ammocharis coranica* (Ker Gawl.) (ground lily) used on tools that experience heavy duty strains and forces. The hunters we observed used iron, not wooden, digging sticks.

## The Preparation of Glue and Adhesive in the Tsumkwe area

### 1. *Ammocharis coranica* glue

**Fig 3:**
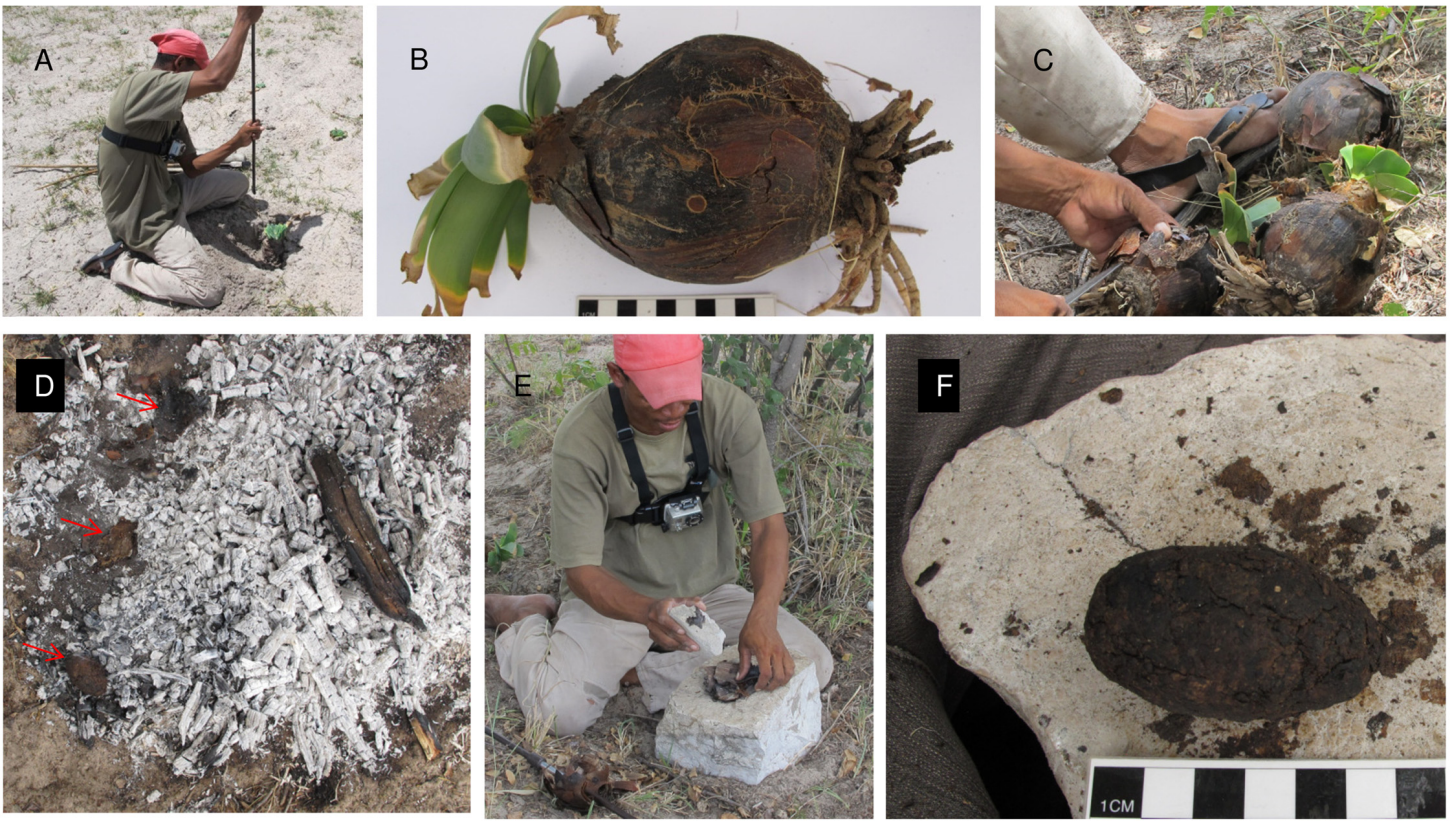
Creating *Ammocharis coranica* glue. A. /uce N≠amce digging the bulb (note the video camera attached to his chest); B. *Ammocharis coranica* bulb; C. Cutting bulb scales; D. Heating bulb scales (marked by red arrows) on coals; E. /uce N≠amce pounding heated bulb scales; F. Glue ball after kneading.

Many *Ammocharis coranica* bulbs grow close to the road between Tsumkwe and Gautscha (S 19. 36.57.1; E 20.30.33.3), in open patches of white sand that might, during heavy rain, become swampy. /uce N≠amce and ≠oma /Kunta set about digging four bulbs (Fig 3A) and they seized them from the soft sand within minutes. The preparations for glue making followed with the collection of twigs, grass and small branches for starting a fire, and *Dichrostachys cinerea* (L.) (sickle bush) branches for creating coals. After lighting the fire, which they rapidly did using traditional fire sticks from their quivers, /uce N≠amce and ≠oma /Kunta found large, flat, calcrete blocks to use as anvils, and smaller calcrete chunks for hammer stones. The men peeled the stiff, brown *Ammocharis coranica* bulb scales (Fig 3B and 3C), using their metal knives. The outer most scales were discarded and only those closer to the fleshy inner core of the bulb were used. Palm-sized scales (or smaller pieces when the scales broke) were removed and piled neatly. The selected bulb scales were then dusted off to remove sand particles. The bulbs themselves were discarded and later replanted at our suggestion. Small coals were scraped to the side of the fire and pieces of bulb scale were placed on these, initially one at a time, later a few at a time, between thirty seconds to three minutes at a time. The scales were turned gingerly, allowed to heat for a few more seconds, then removed and placed on the anvil stone. Here they were pounded and, during pauses, each scale was folded inward on itself. Once softened, the pulp became slightly plastic and its elasticity increased as it was kneaded vigorously with fingers. Scales were constantly added to the fire, a few at a time, so that they could be monitored to stop them from burning. This procedure was repeated and each newly pounded pile of pulp was added to the next. Some ash became incorporated, but this was not deliberate; when ash and charcoal stuck to the scales, they were flicked off, leaving only tiny fragments. Eventually the glue ball resembled a coprolite. The whole ball was reheated briefly on the coals, then kneaded again and declared complete.

The hunters commented that the *Ammocharis* scales were not as pliant as usual and that the glue was reluctant to cohere. Usually the process is easier. They concluded that female bulbs are best for making glue; the ones they dug were considered male. San sometimes refer to different forms or types of plants as male or female, but they also point out that plant properties may be affected by habitat variability. As the glue cooled, it became hard like dried putty. This glue is used as a fixative for the components of large implements or weapons, like axes and spears. It can also be used to repair cracks in clay pots [39], and in Botswana the ground paste is used to waterproof pots [40]. Its pliancy can be restored through reheating. It is not used for arrow production, and it is not stored on the glue stick next to the clear *Terminalia* gum. We did not observe it in use at Eagle Pos (though hunters do use it there), only at Dou Pos.

### 2. *Terminalia sericea* glue

A dense *Terminalia sericea* grove on the Gautscha road (S 19.38.09.9; E 20.30.08.4) was the source of the gum collected. Few of the *Terminalia sericea* trees yielded gum. The men told LW that these were probably female trees, while the many unproductive ones were male. *Terminalia sericea* gum oozes into a honey-coloured nodule that clings to the bark of the tree. This can be picked off. The hunters said that it did not matter whether the gum is dry and brittle, or wet and runny; both types can be used. Dry gum can be powdered and reconstituted with water or spittle, or, as we once observed, the dry gum is simply masticated. ≠oma /Kunta whittled a short glue stick on a straight branch cut from a *Grewia flava* (DC.) (velvet raisin) bush. Opposite the pointed end, he cut a diagonal sliver, so that this ‘platform’ could act as a glue or poison applicator. ≠oma /Kunta chewed a ball of *Terminalia* gum to soften it, and then he moulded it to the lower end of the glue stick. He said that he would use warm water, not spittle, when applying this gum to his arrows. He told us that the gum would take a day to dry on the glue stick.

### 3. *Ozoroa schinzii* adhesive

**Fig 4:**
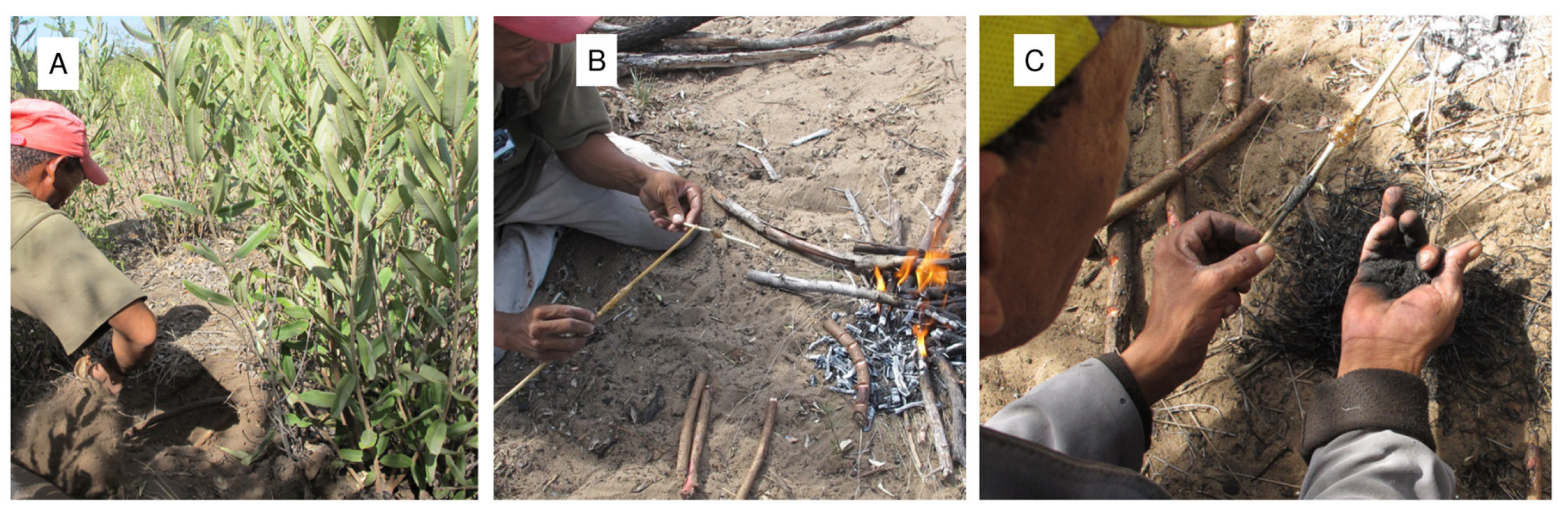
Creating *Ozoroa schinzii* adhesive at Eagle Pos. A. /uce N≠amce digging *Ozoroa schinzii* roots; B. Notched root heated on the embers. /uce N≠amce is using an applicator to smear boiling latex, which oozes from transverse incisions made in the root, on a glue stick; C. ≠oma /Kunta is crushing burnt grass to a fine powder in his right hand, into which he gently presses the *Ozoroa* latex on the stick. Note the lump of *Terminalia sericea* gum on the same glue stick.

**Fig 5:**
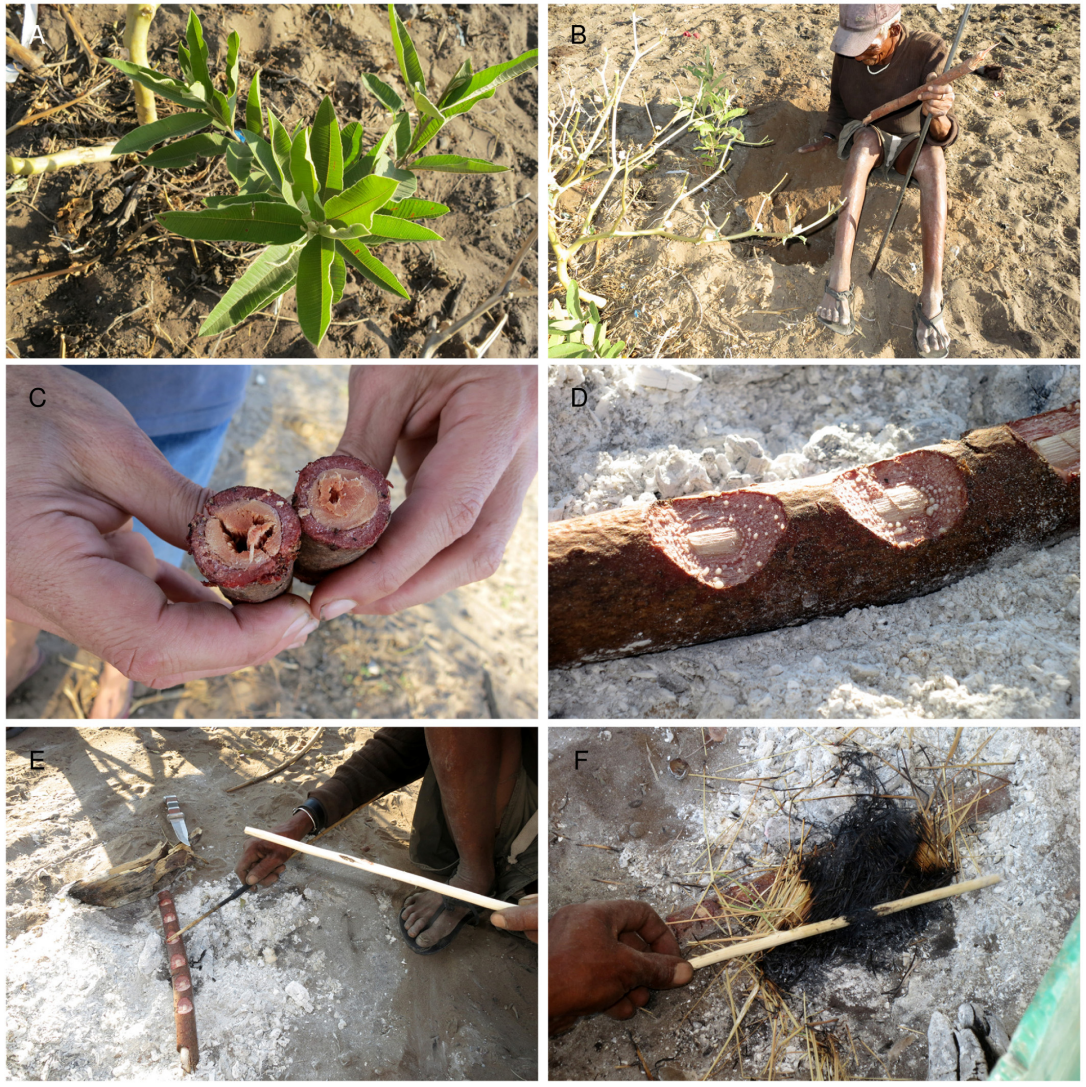
Creating *Ozoroa schinzii* adhesive at Dou Pos. A. *Ozoroa schinzii*; B. //ao ≠Oma holding a metal digging stick and a piece of *O*. *schinzii* root in one hand, while using the other to cover the exposed, damaged root with sand; C. Section of *O*. *schinzii* root showing the difference in colour between the cortex, where the natural latex is located, and the inner area (xylem, phloem, pericycle); D. Drops of latex exuding, during heating, from notches cut into the *O*. *schinzii* root cortex; E. Collection of the latex from the notches; E. Stick with latex is pushed onto burned grass to incorporate charcoal in the latex.

*Ozoroa schinzii* bushes grow near the Eagle Pos village and /uce N≠amce and ≠oma /Kunta swiftly excavated the sand at the base of a clump of bushes (Fig 4A). This *Ozoroa* taxon is a large, rounded, multi-stemmed bush. Five roots were amputated; each stump removed was about 30 cm in length. The holes at the base of the bushes were filled with sand so that the remaining roots would not die. The root bundle was carried back to the homestead together with firewood. /uce N≠amce and ≠oma /Kunta selected a dead *Terminalia sericea* tree and a dead *Combretum* (bush willow) tree and broke off thin branches for firewood. Once the flames were established, the men cut fat, oval slits along one plane of each *Ozoroa* truncheon. Thus notched, the sticks were laid, one at a time, on embers at the edge of the fire (Fig 4B). Almost immediately, a white, milky liquid began to boil in the slits. Once heated, it became reddish-brown like the interior of the root. A previously carved *Grewia flava* applicator was dipped into the latex to transfer it to the glue stick, alongside the *Terminalia* gum. A thick tuft of *Aristida adscensionis* (annual three-awn) grass was collected, set alight and lifted up with a stick to get air through the bundle so that the flame burnt all the way through, carbonizing it in the process. The burnt grass was compressed by hand to form extremely fine, black powder. ≠oma /Kunta gathered the black powder in his palm and pressed it into the *Ozoroa schinzii* latex, turning the stick so that the entire latex ball was well covered (Fig 4). Several more layers of black powder were added and pressed into the latex. //ao ≠Oma from Dou Pos prepared and heated *Ozoroa* truncheons in the same way as the men from Eagle Pos (Fig 5), but he collected the exudate while it was still white. He then pressed the stick with the *Ozoroa* exudate directly into the charcoal powder present on the slab on which he was burning the grass. It appears that there is some variability in the manufacture of this adhesive. After the adhesive was fixed to the wooden applicator, ≠oma /Kunta used his knife to scrape the wood clean between the lumps of fpaste, and along the entire length of the stick. In all the work that the men did, they were meticulous, neat, and paid close attention to fine detail. We were told that the black colour of the *Ozoroa* adhesive was to mark clearly the place where the adhesive was added to the shafts of weaponry, and that without the carbonized grass the exudate is too wet and does not work well. Later we observed that after the *Ozoroa* adhesive was applied to a shaft, sinew was coiled over it, whereas the clear *Terminalia sericea* glue was used on top of the sinew. The *Ozoroa schinzii* latex is said to be a substitute for beeswax and, indeed, the product does not become hard and brittle, instead remaining pliable like wax.

## Using Glue and Adhesive as Part of Making Arrows

**Fig 6:**
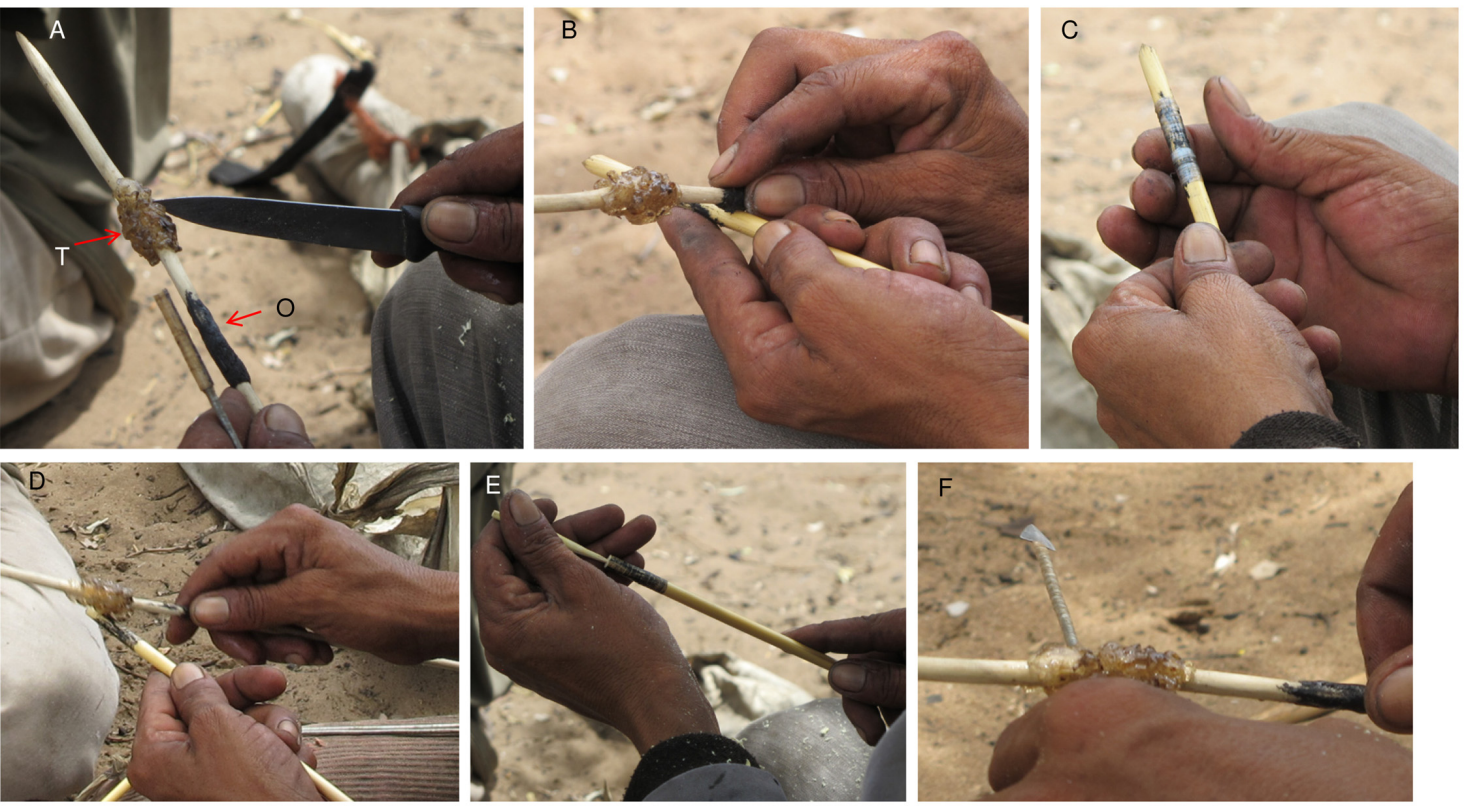
Using glue and adhesive to assemble an arrow at Eagle Pos. A. Glue stick with clear *Terminalia sericea* glue (T) and black *Ozoroa schinzii* adhesive (O); B. *Ozoroa schinzii* adhesive is rubbed on the grass shaft; C. Sinew is wound over the *Ozoroa schinzii* adhesive; D. *Terminalia sericea* glue seals the sinew on the grass shaft; E. The wooden linkshaft is tested for size in the grass shaft; E. *Terminalia sericea* glue seals the sinew on the metal arrow shaft.

**Fig 7:**
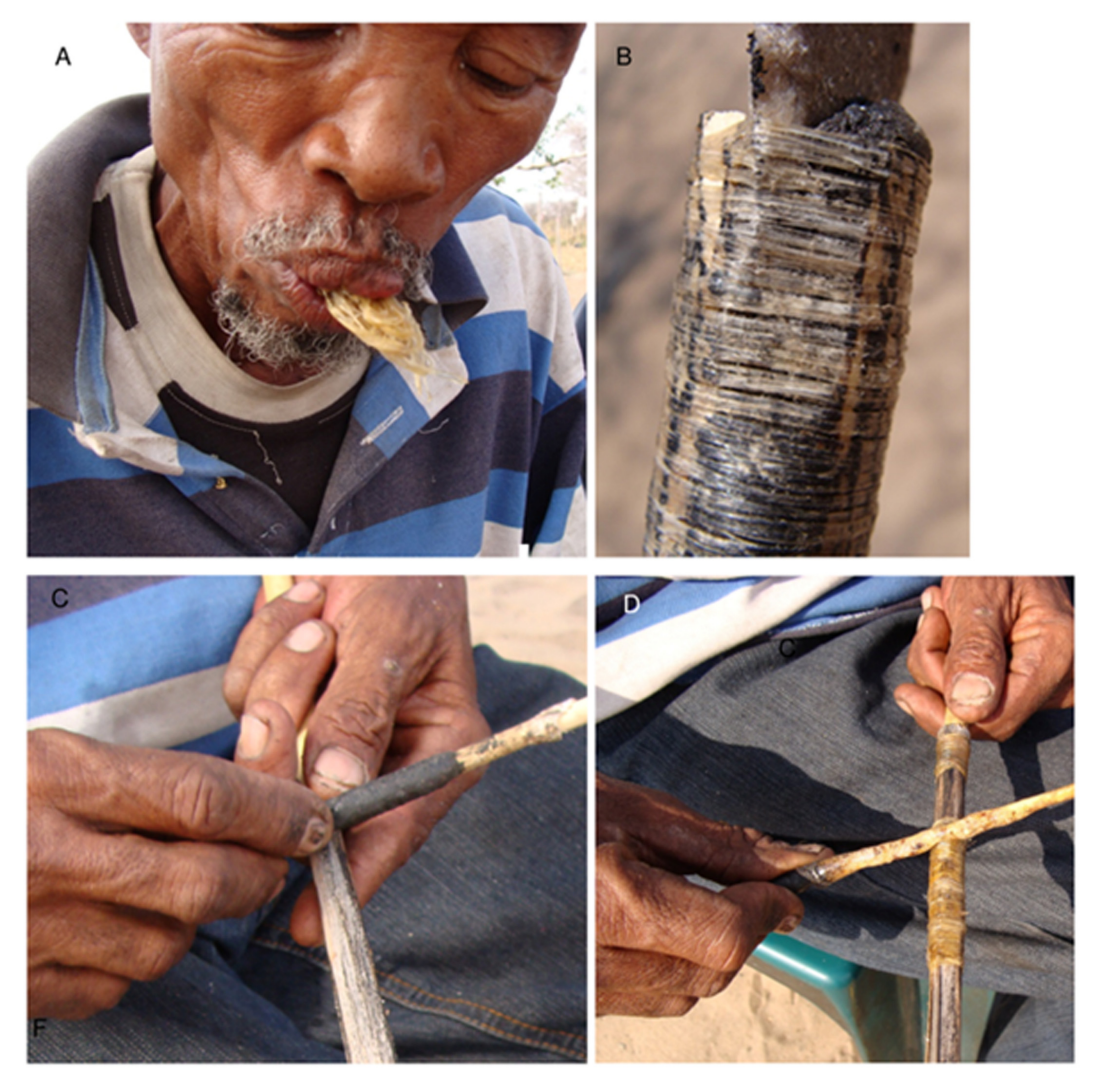
Dengwe methods of processing sinew and using fixative. A. G≠xao Kgao chewing animal sinew to make twine; B. Implement mounted with *Ammocharis coranica* glue, then wrapped with sinew; C. G≠xao Kgao applying *Ozoroa schinzii* adhesive to shaft; D. G≠xao Kgao applying *Terminalia sericea* gum to sinew on shaft.

**Fig 8:**
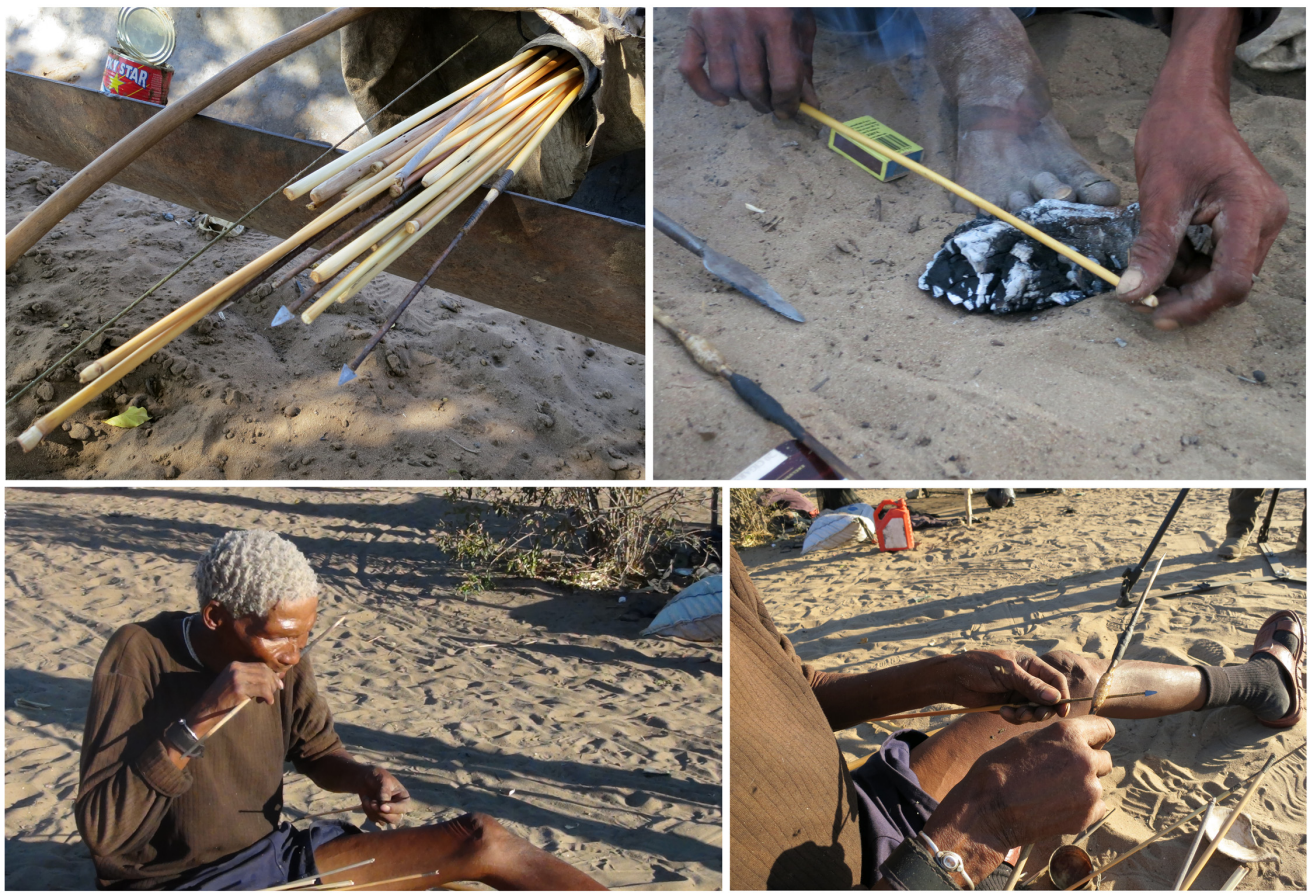
Preparing hunting equipment at Dou Pos. A. Hunting bag with quiver containing complete arrows with arrow shafts covered with dried poison, unused arrow shafts, and wooden sticks ready to be transformed into poison applicators; B. Arrow shaft straightened by pressing and rotating on a piece of large, dying charcoal; C. //ao ≠Oma licking dry *Terminalia sericea* gum attached to a glue and poison applicator; D. Application of *T*. *sericea* gum on sinew covering the metal arrow shaft.

**Fig 9:**
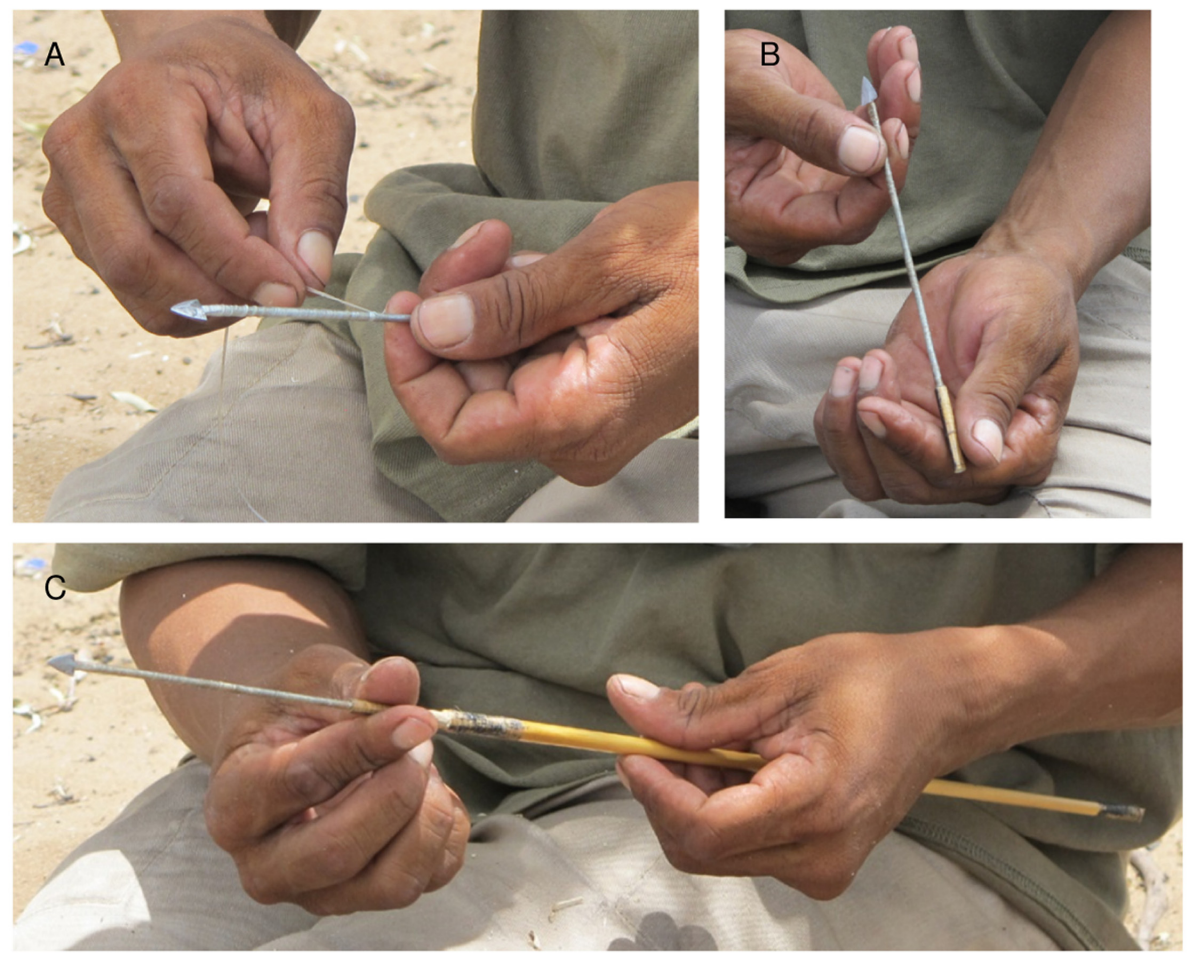
Preparing a metal arrowhead. A. Sinew is bound to the metal shaft and glued with *Terminalia sericea* glue; B. A grass collar is glued to the metal shaft with *Terminalia sericea* glue; C. The complete arrow is attached to the grass shaft with a wooden linkshaft. The linkshaft is glued to the grass collar so that the three component arrowhead is a single unit, whereas the grass shaft is loose and will detach during the hunt.

Traditionally arrowheads used in the Nyae Nyae area were manufactured from bone, but iron arrowheads have largely replaced these. We saw ≠oma /Kunta and /uce N≠amce fashion two iron arrowheads from short pieces of fencing wire that they beat on a metal anvil, using a metal hammer with a short handle. These arrowheads were to be mounted on shafts of blue grass culms (*Andropogon gayanus*) and fixed with *Ozoroa schinzii* adhesive and *Terminalia sericea* glue. Arrow manufacture has been recounted elsewhere (see, for example, [33,34]), so here we concentrate on describing the use of the fixative pastes during weapon manufacture. While the metal arrow tips were being shaped and filed, a strip of kudu sinew soaked in tepid water in a tin mug. ≠oma /Kunta emptied his quiver and selected two new blue grass shafts. He checked whether they were straight and he was not satisfied so he scraped coals from the edge of the fire and laid each shaft on the hot sand for a few moments. //ao ≠Oma from Dou Pos straightened his shafts directly on a large dying coal. The shaft is manipulated to straighten it, then checked repeatedly by sighting down its length. With this quality control complete, ≠oma /Kunta held his glue/applicator stick (Fig 6A) firmly and rubbed waxy, black *Ozoroa schinzii* adhesive to about three centimetres of each end of the shaft (Fig 6B). Kudu sinew was then stripped into millimetre-wide lengths of 20 to 25 cm. ≠oma /Kunta pulled a sinew thread through his mouth to stretch and straighten it against his teeth, then deftly wrapped both ends of the shafts to cover the area marked black by the adhesive (Fig 6C). G≠xao Kgao from Dengwe chewed a wad of sinew before using it (Fig 7A). The black *Ozoroa schinzii* adhesive held the sinew in place on the shafts. ≠oma /Kunta left the last centimetre of shaft unwrapped at both ends, and later he cut one end off to neaten it. A nock to accommodate the bowstring was cut into the other end. The binding prevents the shaft from splitting. The *Terminalia* glue lump was moistened with water from the mug, and rubbed on the sinew to seal it to the shaft (Fig 6D). More sinew strips were prepared, and the entire length of the metal shaft of the arrowhead was then wrapped with sinew. *Terminalia* glue was rubbed over these sinew bindings to seal them and to provide a secure foundation for the poison that would soon be applied. The same process was followed at Dengwe (Fig 7) and Dou Pos, where //ao ≠Oma licked dry *Terminalia* glue before using it (Fig 8). The *Terminalia* glue on the base of ≠oma /Kunta’s metal shaft was designed to fasten it within the short grass collar that he made next (Fig 9B). He took a scrap of blue grass culm from his quiver, whittled it a little, bound it with sinew, and slipped it onto the metal shaft. He twisted the arrow until it fitted snugly, then cut off the end of the collar, leaving only a short hollow section exposed to accommodate a linkshaft. He selected a straight branch from a nearby *Grewia flava* bush and gave half to /uce N≠amce. The men whittled their double pointed linkshafts, and used them to connect the grass shafts and metal arrowheads. The linkshaft was adjusted and readjusted until the fit was perfect (Fig 9C). Balance and straightness were repeatedly checked. Then *Terminalia sericea* glue was applied to one end of the linkshaft to be inserted into the collar, and glue was now applied within the hollow itself. The three component arrowhead thus became a single, inseparable unit (Fig 9C) that would detach from the grass shaft when the arrow met its target. Finally the arrow was ready for poisoning.

## The Preparation of Poison for Use on Arrows

Poison production is complex and involves planning, many action steps, and considerable care and skill. Since hunting gear has associated symbolism [34] the poison recipes may also have symbolic meaning. We do not, however, explore that theme here. The hunters were unerring in their judgment of where a particular product would be found, even when this was more than 30 km from their village. There is no single poison recipe, but the most important ingredient, common to all, is the grub of a Chrysomelid beetle found deeper than 30 cm below the ground, buried under infected *Commiphora* (corkwood) spp. or *Sclerocarya birrea* (marula) trees that serve as their host plants. These beetles can also be parasitized by *Lebistina* beetles, which are said to be more toxic than their hosts [41]. The toxic principles have been chemically analysed [42–45]. Woollard and colleagues [43] conclude that *Diamphidia* pupae contain a protein toxin that is responsible for its lethal properties. The poison was once thought to be neurotoxic, but Kao and colleagues [44] found no evidence for this and propose that prey deaths can probably be attributed to hemolysis and generalized tissue hypoxia.

**Fig 10:**
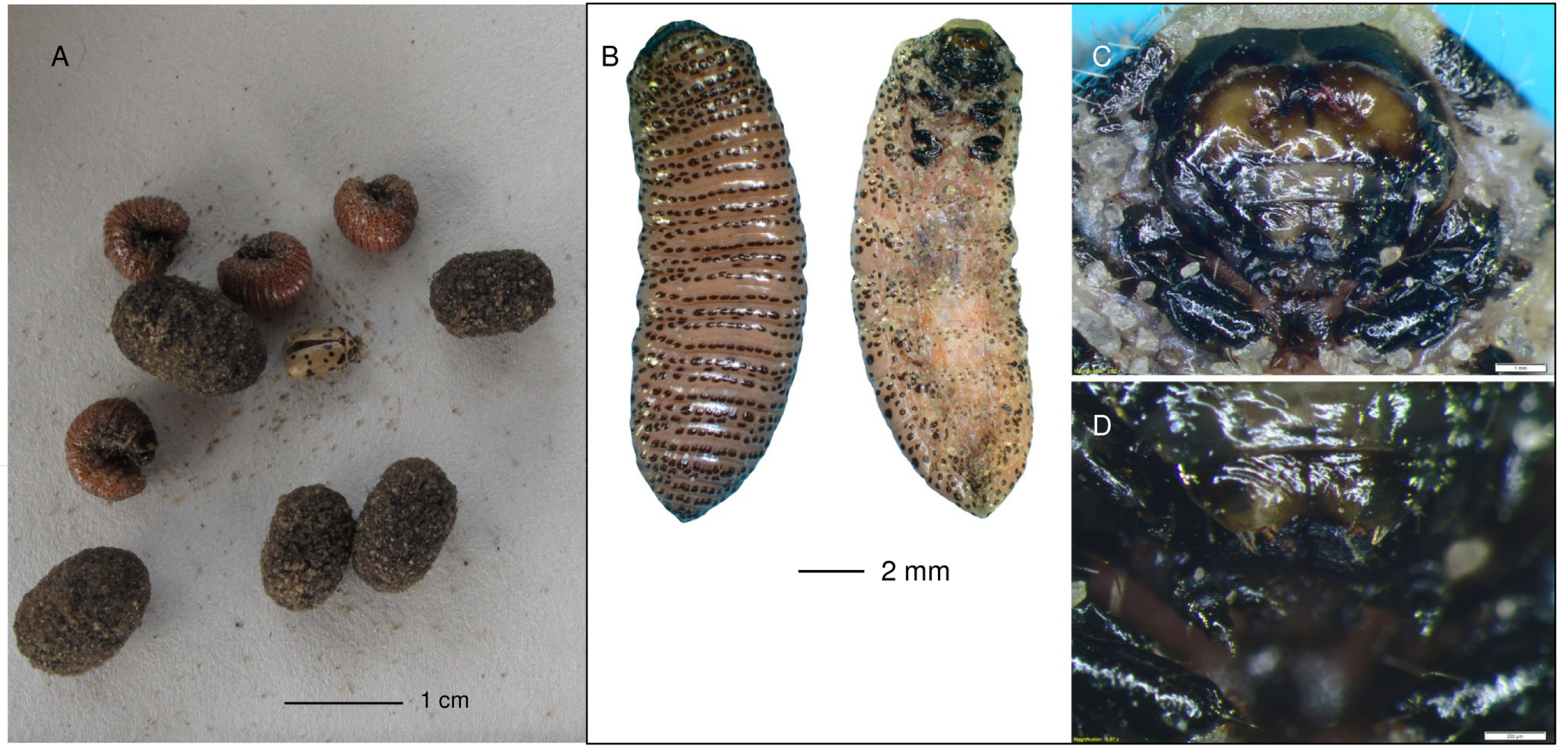
Three stages in the life cycle of the Chrysomelid beetle found under a marula tree in Tsumkwe. A. the gold and black-spotted beetle, pink, naked grub and earthen cocoon containing the grub; B. *Polyclada* sp. grub, dorsal and ventral views; C. *Polyclada* sp. grub mouthparts. Scale 1 mm; D. *Polyclada* sp. grub mouthparts. Scale 200 μm.

*Diamphidi*a spp. and *Polyclada* spp. occur in the Tsumkwe area, and both genera can be hosted by either corkwood or marula. Identifying the genera and species is not straightforward from the grub for there are many morphological similarities amongst Chrysomelids [46,47]. However, the grubs that ≠oma /Kunta and /uce N≠amce collected have ornately spotted skin (Fig 10) and are therefore *Polyclada* sp. We targeted marula trees in Tsumkwe (S 19.36. 02; E 20. 30. 08.4) and ≠oma /Kunta and /uce N≠amce dug around tree roots with their iron digging sticks. The hunters dug carefully in order to minimize crushing the *Polyclada* sp. grubs and in so doing rendering them useless. Sometimes the men created cavities in the sand to undermine the sediment above, causing small cave-in effects. The men felt for lumps in the sand and rubbed these gently between their palms so that they could see whether oval, earthen cocoons (Fig 10) were hiding in the damp sand.

Not all trees are infected, but when one of these is located, several dozen cocoons can be extracted from between its roots. Notwithstanding the inherent deadliness of Chrysomelid grubs, the Tsumkwe hunters add several other ingredients to strengthen or complement the poison. The stated purposes of these ingredients will be explained shortly. The collection and extraction of the additives is time consuming and intricate. We were told by /ui G/’aqo, G≠xao Kgao, //ao ≠Oma, /uce N≠amce and ≠oma /Kunta about the following additives: *Asparagus exuvialis* (Burch.) (wild asparagus) sap, *Acacia mellifera* (Vahl.) (black-thorn acacia), *Harpagophytum procumbens* (Burch.) (devil’s claw), and *Swartzia madagascariensis* (Desv.) (snake-bean), but others to be mentioned in the Discussion are also listed in Giess and Snyman’s notes [48]. Giess was a botanist from the Windhoek Herbarium and, in the 1960s, he collected plants and information about their uses in the Tsumkwe area.

We now describe the collection, preparation and uses of the additives for the poison.

### 1. *Asparagus exuvialis* sap

**Fig 11:**
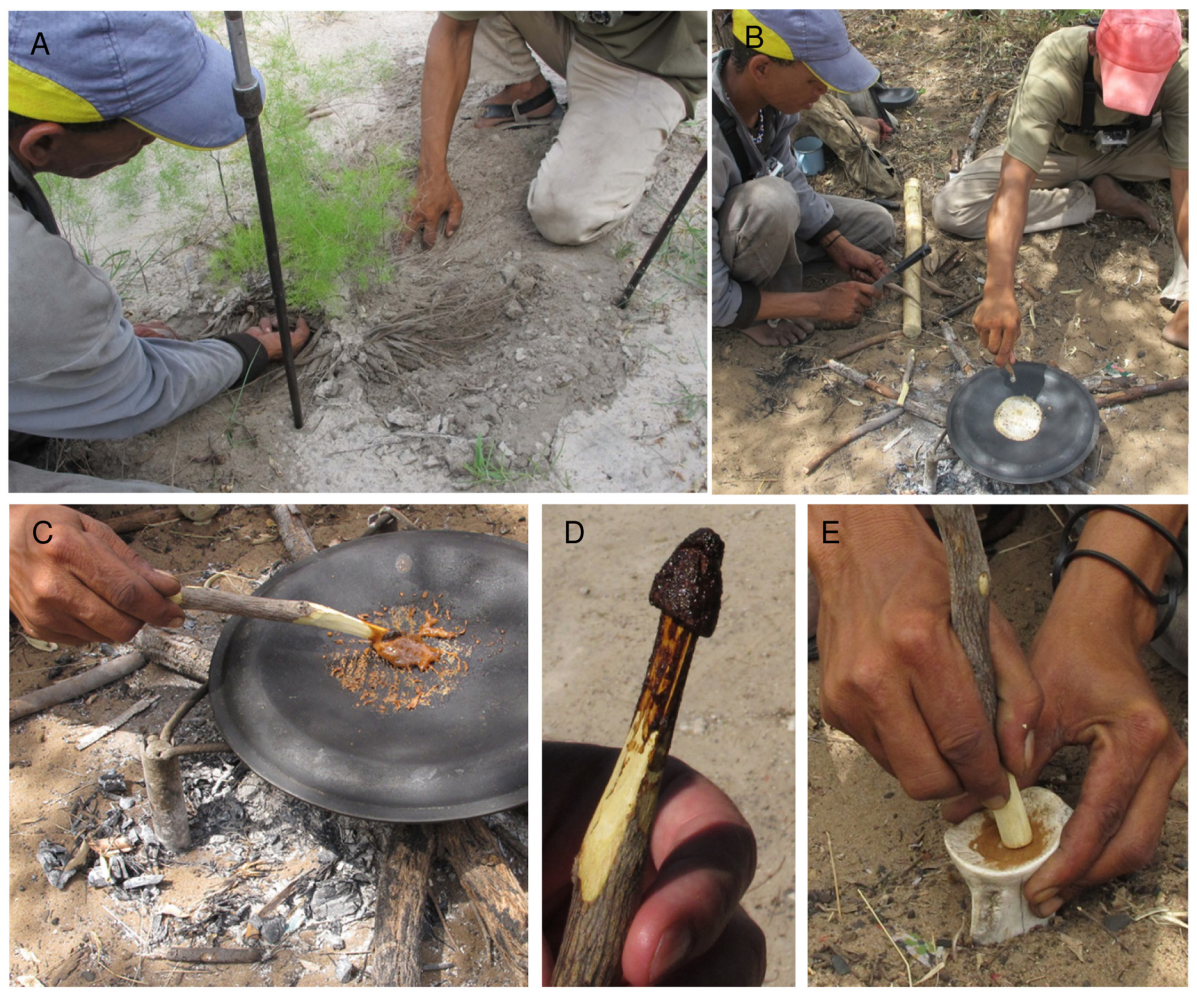
The collection and preparation of *Asparagus exuvialis* additive at Eagle Pos. A. ≠oma /Kunta and /uce N≠amce digging *Asparagus exuvialis* roots; B. ≠oma /Kunta softens a root by tapping it, /uce N≠amce squeezes sap on the pot lid; C. The heated *Asparagus* sap is stirred until it reduces and darkens; D. Hardened *Asparagus* sap on the stirring stick; E. Grinding the hardened *Asparagus* sap to powder in the glenoid cavity of a scapula mortar.

On the road to Gautscha, only a few kilometres from Tsumkwe (S 19. 37. 50.6; E 20. 31. 17), we found the *Asparagus exuvialis* plant that the hunters use to add to the grub poison. Its foliage is soft and delicate, with no spines, on a central, zigzag stem, from which white cuticle peels. The bush is less than 50 cm high, with a rosette of tubers like mini sweet potatoes. In the village of Dou Pos, //ao ≠Oma said that this is the female *Asparagus* plant and that the male plant has spines and looks different. /uce N≠amce and ≠oma /Kunta dug the *Asparagus* tubers to expose them (Fig 11A), picked ten, then covered the remaining tubers with sand so that the plant would live. Back at Eagle Pos, the *Asparagus* tubers were emptied from ≠oma /Kunta’s hunting bag. A cast iron pot lid was inverted on a low, metal tripod over the fire. Each *Asparagus* tuber was beaten firmly, but gently, with the back of a knife (Fig 11B). When softened, juice was squeezed like toothpaste from each tuber onto the heated lid (Fig 11B). The liquid was agitated with a freshly cut spatula of *Grewia flava;* the tubers were squeezed one at a time and the clear liquid was mobilised energetically. Colour changed to brown, then dark orange-brown, as it boiled and reduced (Fig 11C). Eventually it became toffee-like and stuck to the stick (Fig 11D). It was then removed from the pot lid, cooled, and stored on the stick in ≠oma /Kunta’s quiver. The heated, reduced *Asparagu*s sap is powdered (Fig 11E), then added to the grub poison to stop the animal that is shot from urinating. This makes the poison act more quickly than it would without the *Asparagus*, because the affected animal cannot eliminate poison from its body. If, despite the medicine, the animal does urinate, only a small amount of blackish urine is expressed, and this helps the hunters to track the wounded animal amongst the spoor of the herd.

**Fig 12:**
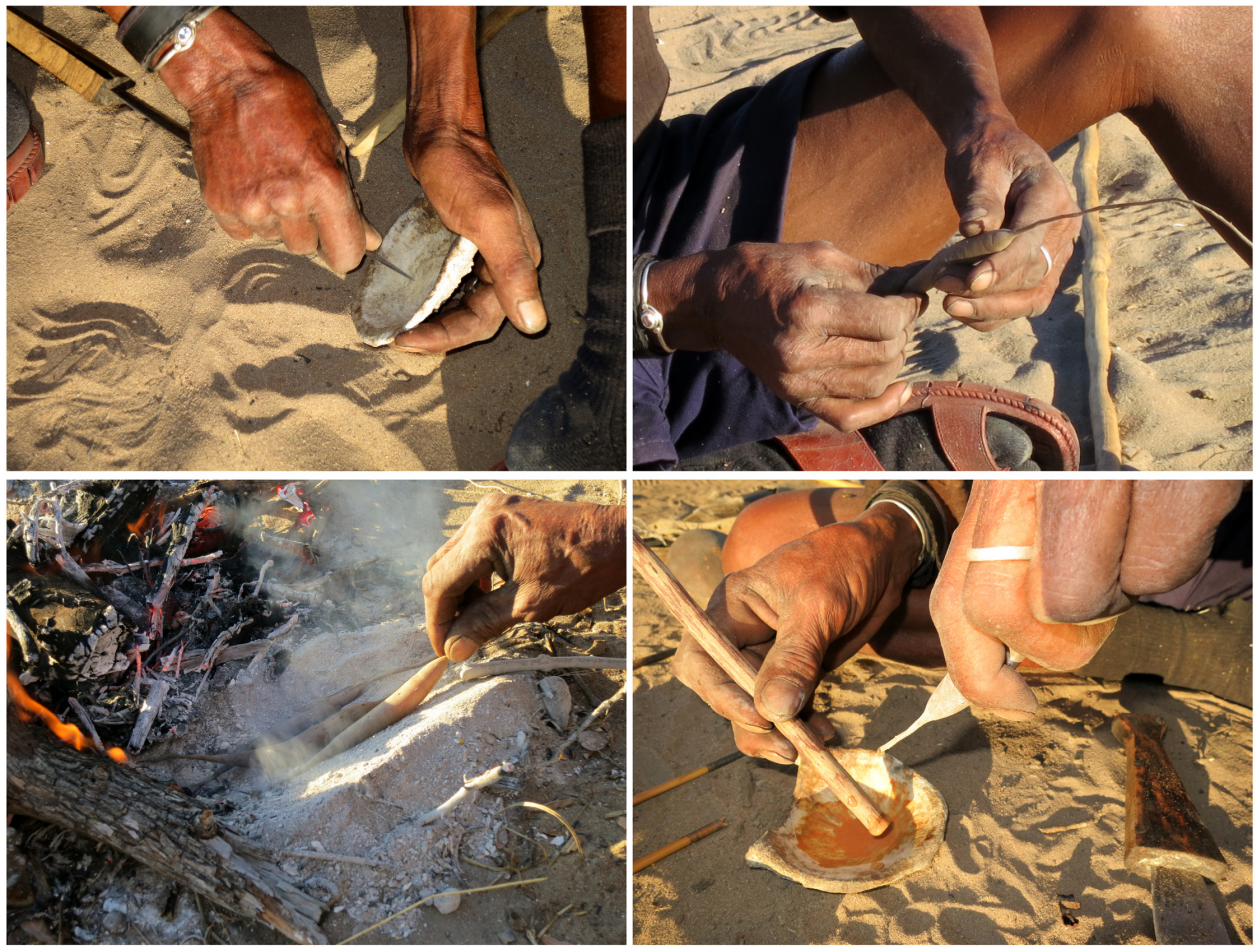
Preparing arrow poison at Dou Pos. A. //ao ≠Oma cleaning the articular facet of a giraffe vertebra to be used as a container for mixing poison; B. //ao ≠Oma squeezing *Asparagus exuvialis* tuber; C. Heating the *A*. *exuvialis* tubers in hot ashes; D. Squeezing sap from the tuber into the vertebra containing poison grub viscera.

//ao ≠Oma from Dou Pos used a different method to process *Asparagus exuvialis* (Fig 12). First, he carefully cleaned the concave surface of the articular facet of a giraffe vertebra to remove old, dry poison. He told us to move away from the direction of the wind to avoid getting poison residues in our eyes. Secondly, he squeezed the entrails from three larvae into the vertebra and mixed the orange substance (fat and bodily fluids) with the end of a stick. Then he squeezed the *Asparagus* tubers in his hands, to facilitate the release of the liquid inside, and heated the *Asparagus* tubers on hot ashes. When the tubers were hot, he took them out and gently hit them one by one with a stick. After this he broke the thin end of each tuber and squeezed it, extracting drops of transparent liquid that he mixed with the larvae viscera in the vertebra. The *Asparagus* liquid rendered the mixture thinner than before. This mixture was put on the metal shaft covered with sinew and glue, and then gently heated to fix the poison on it.

### 2. *Harpagophytum procumbens* tuber liquid

On the road to Gautscha, near to where we previously gathered *Terminalia sericea* gum (S 19. 37. 46.9; E 20. 31. 14.7), ≠oma /Kunta and /uce N≠amce collected *Harpagophytum procumbens* secondary tubers from around the sides of the small plant that rambled inconspicuously on the sand. The tubers are fatter, longer and harder than *Asparagus* ones, and they are a yellow-brown colour. The tubers, better known for their arthritis-relieving powers [49], are used as an additive in hunting poison when wild asparagus or snake bean are not available. /uce N≠amce said that preparation of *Harpagophytum procumbens* entails creating a pulp from the tubers, then squeezing the juice directly into the poison cup. Cwi G|'aqo mentioned another technique involved scraping the skin off the tubers, then boiling them until brown and thick, before adding the concentrated liquid to the bone mortar. Since *Asparagus* was available on this occasion, ≠oma /Kunta and /uce N≠amce did not add *Harpagophytum* to their poison mixture. The *Harpagophytum*, like the wild *Asparagus*, stops the animal from urinating, or makes urine black so that the animal can be easily tracked.

### 3. *Acacia mellifera* bark extract

Near Eagle Pos, /uce N≠amce chopped the bark from an *Acacia mellifera* tree and ≠oma /Kunta peeled off the outer layer of bark and discarded it, keeping coils of the cream-coloured inner bark, which he placed in his hunting bag.

### 4. Powder ground from roasted *Swartzia madagascariensis* pod

The snake bean was not required for the composite poison recipe that /uce N≠amce and ≠oma /Kunta used, but /uce N≠amce demonstrated its preparation. He buried the bean in the embers of a cool fire for approximately three minutes, pulled the roasted pod from the coals, cooled it briefly, then scraped the charred pod to remove the outer shell and harvested the roasted inner flesh, but not the seeds. Roasted inner flesh from the pod is ground to powder and mixed with the Chrysomelid grubs. This information is supported by Giess and Snyman [48] who add that the snake bean powder is thought to enhance the concentration of the poison. The fruit pods of *Swartzia madagascariensis* are known to contain saponins [50]. However, the snake bean is not local to the Tsumkwe area, and the two pods which /uce N≠amce and ≠oma /Kunta showed us at Eagle Pos were gifts from people in the N’homa and Samangaigai area. The snake bean used by the Dengwe hunters was also brought from elsewhere.

### 5. Combining ingredients to create composite poison

**Fig 13:**
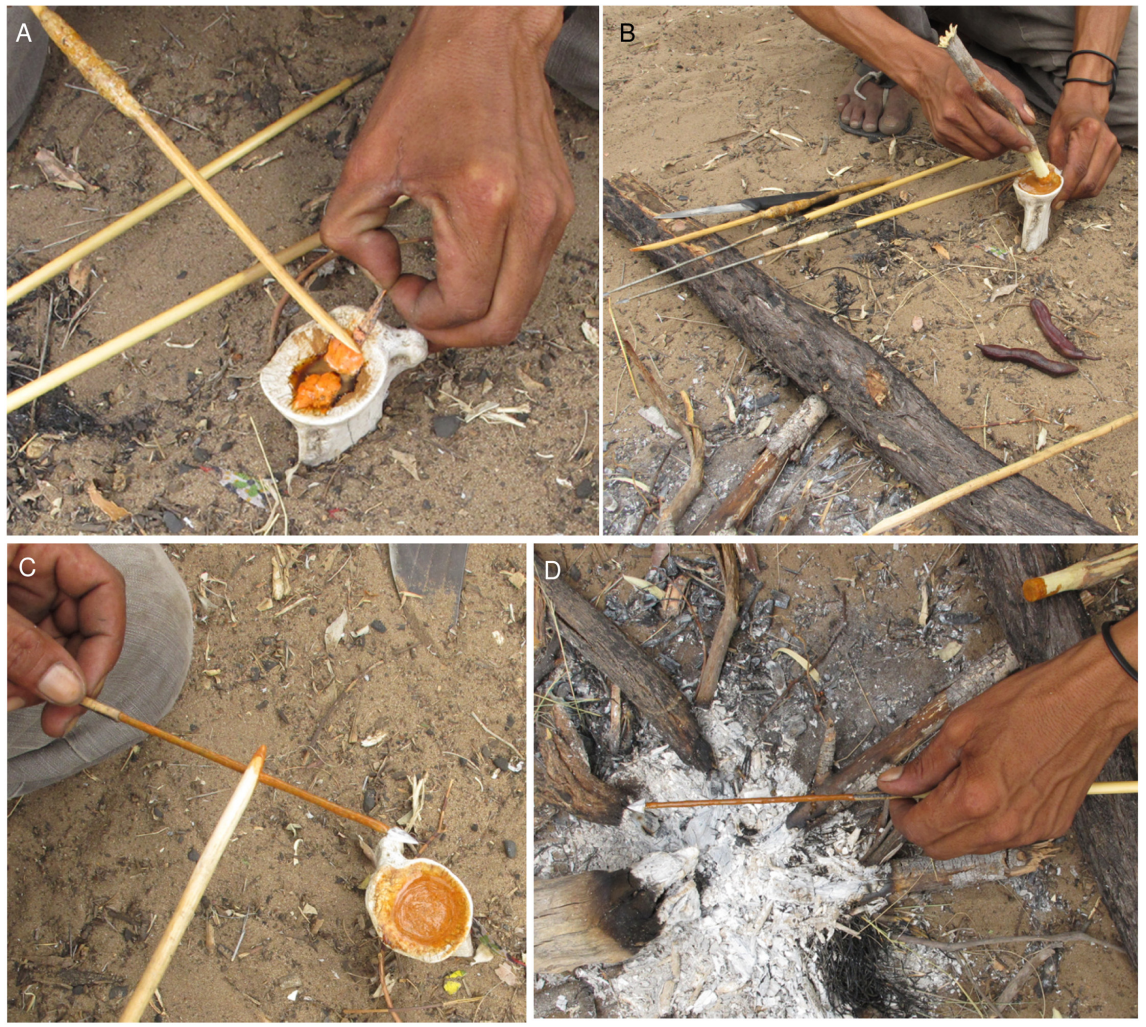
Making composite poison for arrows at Eagle Pos. A. Squeezing grub viscera onto the *Asparagus* powder in the scapula mortar; B. After adding *Acacia mellifera* inner bark and spittle mix, the poison is mixed well in the mortar. Note the snake beans in the foreground; C. Poison is applied to the sinew on the shank of the arrowhead using a wooden applicator; D. The poisoned arrow is twirled over the coals to dry the poison.

The men rekindled their Eagle Pos hearth with a few *Terminalia sericea* sticks, and lit a tiny fire. A glenoid cavity of a kudu scapula stood upright in the sand near the fire. This concave bone was to be the mortar and mixing bowl for the arrow poison. ≠oma /Kunta cut a blunt ended *Grewia flava* stick, about 25 cm long, to use as a miniature pestle. First, ≠oma /Kunta ground a few grams of the *Asparagus* exudate that he scraped from the storage stick. To this fine reddish-brown powder, both men warily added the viscera from the Chrysomelid grubs. The men worked cautiously with these grubs as even a small amount of their bodily fluids entering the bloodstream through tiny wounds on their hands could be fatal, with no effective antidote [33]. The heads of the poison grubs were nipped off and the bright orange, fatty innards as well as a clear bodily fluid were squeezed onto the powder in the scapula mortar using a wooden applicator (Fig 13A). GT observed that in one grub alone, about ten drops of this translucent liquid came out. Eleven Chrysomelid grubs were added to the scapula mortar, although the hunters stated that they would usually add seven or fourteen, depending on the number of arrows being produced. Each grub skin sheath was discarded in the fire to destroy it, and ≠oma /Kunta even took care to discard the decapitated heads in the fire. The Chrysomelid grub-*Asparagus* mixture was guardedly stirred with the blunt pestle stick (Fig 13B). Then ≠oma /Kunta chewed a length of *Acacia mellifera* inner bark and spat the yellowish liquid from the pulp into the mortar. *Acacia mellifera* makes the poison stronger, but it also acts to service and curate the arrows, keeping the compound from drying out and flaking off the metal arrowhead. Leon stated that when *Acacia mellifera* liquid is added to the poison, it foams like soap. This causes the victim to foam from the nose and mouth, further hastening its death as it struggles to breathe.

The composite poison was now ready to apply to the arrows. Each man took one arrow. The poison applicator was used to lift and smear the orange poison entirely along the length of each sinew-wrapped arrow shank, cautiously avoiding the sharp arrowhead as well as fingers (Fig 13C). The application was slow and repetitive, and nothing was spilt on the ground. Anointed arrows were twirled just above the dying coals to dry the poison (Fig 13D). A log was placed near the coals and the arrows were then propped upright against it, their bases pushed into the sand. The poison thus dried slowly next to the fire. While the poison was drying, both men used their knives to whittle away traces of poison from their applicator sticks and the wooden pestle. The scapula mortar was wiped clean with the chewed pulp of the *Acacia mellifera* inner bark, and afterwards with a slice of *Harpagophytum procumbens*; then both cleaning agents were discarded next to the fire.

/uce N≠amce took the opportunity to renovate an old arrow that he took from his quiver. He scraped from the metal shaft the existing, dry and cracked poison and flaking sinew, then chewed a length of kudu sinew, wound it the full length of the metal shaft, and rubbed the sinew wrapping with *Terminalia sericea* gum moistened with saliva. This renovated arrow was also poisoned.

## Discussion and Conclusions

The Ju/’hoansi of the Tsumkwe area in the Nyae Nyae Conservancy demonstrated for us the manufacture and use of traditional fixative pastes and poison recipes. Each paste plays a task-specific role, and none of these fixatives can be a substitute for the others. We did not observe any differences in the manufacture or use of paste ingredients in the three villages. *Ammocharis coranica* bulb scale glue is a simple, single component, waterproof fixative paste that can be reconstituted by heating and kneading. It is used for fixing heavy duty weapons (like spears) and implements (like axes) to their shafts or handles, and for mending various artefacts. *Ozoroa schinzii* latex is mixed with powdered carbonized grass, *Aristeda adscensionis*, to form a compound adhesive that remains pliable and is not water soluble. Beeswax can be a substitute for *O*. *schinzii* latex, but we did not see wax in use. There is a decline in bees in the region according to Tsamkxao ≠oma (Chief Bobo). The *Ozoroa* adhesive is the first fixative smeared on the grass arrow shaft to enable binding with animal sinew to prevent the shaft from splitting. *Terminalia sericea* gum is simple, water soluble glue (a saccharide) that is used for the final sealing of sinew on spear shafts or on arrow components such as grass shafts, collars and metal arrow shanks. Silberbauer [35] reported the use of *T*. *sericea* glue by G/wi, near Ghanzi in Botswana. Some plant use practices are therefore widespread and cross language barriers.

In Nyae Nyae, Chrysomelid beetle grubs are the most important component of modern arrow poison, but several plant taxa serve as optional additives to the grub entrails. The choice of plant, and means of preparation, can vary between villages. To some extent this variability among poison recipes is due to availability. The snake bean is, for example, in short supply in parts of Nyae Nyae, and people rely on gifts of *Swartzia madagascariensis* pods from those with access to the trees. All three villages we visited used *Asparagus exuvialis* tuber sap mixed with grub entrails, but its preparation process varied. Eagle Pos hunters boiled and reduced *A*. *exuvialis* tuber sap, powdered the hard product, and then combined it with poison grub viscera from *Polyclada* sp., and saliva from the masticated inner bark of *Acacia mellifera*. In contrast, the Dou Pos hunters blended viscera with fresh, heated sap squeezed directly from *A*. *exuvialis* tubers. In the use of these two recipes we see both the continuity of traditions, and people’s flexible adaptation to new circumstances. The old man, //ao ≠Oma, may have retained the traditional way of processing *A*. *exuvialis* tubers, whereas the younger hunters at Eagle Pos adapted the traditional method for use with store-bought equipment. Archaeologists are unlikely to recognise such subtle differences in preparation of ingredients.

Furthermore, the application of the poison to the sinew-wrapped shanks of metal arrowheads was done in the same way at all three villages; it is the finale to a time consuming and activity-rich process of product extraction and preparation. Even though we have recorded some minor differences in poison recipes between close villages, the weapons themselves seem standardised in form and manufacture. Traditional Ju/’hoan San social organisation is, in part, responsible for enabling regional uniformity in technology. Standardisation of hunting technology is facilitated by fluid band membership, systems of gift exchange [51], a culture of sharing [33], and a ‘humility ethic’ [34]. Furthermore, hunting customs in the Nyae Nyae area traditionally did not require hunters to restrict themselves to the territory of their own band [33]. Although social change has been inevitable following the introduction of amenities such as houses, schools and clinics [37], much of Ju/’hoan technology remains similar to that recorded by anthropologists sixty years ago. Yet, notwithstanding the tendency for technological conformity, the hunters adapt readily to the use of new raw materials when these offer convenience or improved performance. The employment of metal arrowheads instead of traditional bone points is the best available example, but the recent replacement of organic quivers with plastic pipes is another case in point.Weaponry manufacture was described by Marshall [33] and Lee [34] in sufficient detail that we can conclude that Ju/’hoansi today have weapon components essentially the same as those used fifty years ago. Wiessner [51] comments that there are no regionally specific stylistic features in their arrow points; all Ju/’hoansi use triangular metal arrowheads. In contrast, G/wi and! Xo have tangs on their triangular arrowheads, so these geographically distinct linguistic groups are also separated stylistically [51]. Intimate botanical knowledge and knowledge of raw materials in the Nyae Nyae environment are learned during childhood because children watch hunters making and refurbishing weaponry. It takes many years for a man to become an accomplished bow and arrow hunter. Boys play at shooting with toy arrows from about the age of seven onwards until, at adolescence, they begin to hunt with their fathers [33]. With rare exceptions, all men hunt and there is social pressure among men to be hunters [33]. Men make and poison their own arrows even though men may share batches of poison, take turns in making it [33, 34], and sometimes exchange arrows [51]. The strength of the poison mixture is critical to the success of the hunt because the arrows are adapted to transport poison, not to kill animals by wounding them [33, 34]. The materials science involved in making the poison allows some leeway for experimentation in terms of additives, but the poison grubs seem to be essential ingredients. The Nyae Nyae hunters distinguish *Diamphidia* and *Polyclada* genera, but they consider them male and female of the same taxon and will use either [33, 34].

We look, now, at a few earlier records of hunting equipment, glue and poison used by the Ju/’hoansi and by other hunter-gatherers in southern Africa. Marshall [33] wrote about the use of *Andropogon gayanus* grass shafts and an unspecified black plant paste for sealing sinew to shaft. She furthermore recorded the use of yellow gum, from an *Acacia* tree (whereas we observed the use of *Terminalia sericea* gum), for gluing sinew to the metal arrow shank [33], but she did not document that the black adhesive forms the first layer of fixative paste on shafts, and that yellow gum is the final, outer sealant. She chronicled the four components of the deadly arrow, the use of poison grubs and the mixing of composite poison [33]. She confirmed that various recipes are used, depending on the availability of products. In the 1950s she observed ≠oma Tsamkxao (Leon’s grandfather) making poison in a knee joint bowl, using grub viscera and slivers of a pod that he called! gaowa. This Ju/’hoan name is verified by Giess and Snyman [48], and we therefore know that that old ≠oma Tsamkxao had used the snake bean, *Swartzia madagascariensis*. In addition, old ≠oma Tsamkxao added liquid from roasted *Sansevieria* leaves to his potion and this was said to introduce a fixative paste, not extra poison [33]. The Dengwe hunters we met also made use of *Sansevieria* leaves (Fig 2), although they used them for twine, not poison. Giess and Snyman’s notes [48] suggest that the plant used is most likely *Sansevieria aethiopica*. In a separate poison-making session, Marshall [33] recorded the use of an *Asparagus* sp. root, called n/i!go, to strengthen the poison. Giess and Snyman [48] identify this particular *Asparagus* as *A*. *exuvialis*, and they were told, as we were, that the additive stops the wounded animal from urinating.

Lee [34] has clear illustrations of the four Ju/’hoan arrow components, but his only mention of composite arrow poison is a comment that the gum of several *Acacia* species may be mixed with saliva and then with the poison to improve its adhesive qualities [34].

Giess and Snyman [48] confirm the use of poison recipe ingredients that we heard about at all three villages in Nyae Nyae, and they list some that we were not told of. Potential additions to the poison grubs include: *Ipomoea bolusiana*, *Jatropha erythropoda*, *Lonchocarpus nelsii*, *Raphionacme burkei*, *R*. *lanceolata*, *Sansevieria aethiopica*, *Solanum kwebense* and *Tarchonanthus camphoratus*. *Ipomoea bolusiana* and *Jatropha erythropoda* tubers are grated and the juice that is squeezed out is used to mix into arrow poison [48]. Chewed *Lonchocarpus nelsii* bark is said to soften the arrow poison directly before hunting [48], while *Raphionacme lanceolata* juice from the tuber may be applied to the poison to keep it firmly attached to the arrow [48], and liquid from roasted *Sansevieria aethiopica* leaves or *Tarchonanthus camphoratus* leaf tea serves the same purpose [48]. Four or five fresh pupae of Chrysomelid beetles are macerated with a few grams of powder from baked *Swartzia madagascariensis* seed pods and a salivary extract of *Acacia* bark [52].

Some of the plants described here are environment specific and will therefore not be available for collection in different southern African biomes. Poison grub manifestation is restricted to particular vegetation communities because the beetles are hosted by trees in the Anarcadiaceae and Burseraceae families. Surveys suggest that there are at least 25 poison recipes known in southern Africa [15,53]. Both Schapera [54] and Dornan [55] record recipes in which plants can be used alone, as well as recipes in which plant and animal components (for example, snake poison as a substitute for the poison grub) are mixed. Early travellers in the 1700s, such as the botanist and medical man, Thunberg, mentioned the use of poison by all hunter-gatherers that he encountered across southern Africa [56–58]. Baines [59] witnessed the use of poison grubs (‘Kaa) on arrowheads in 1861, but did not observe the mixture of plants with grubs. He commented, however: “….it seems that every tribe has its peculiar recipe, and herbs and roots are used extensively by some of them.” [59]. This underscores the likelihood that poison recipes are part of cultural traditions backed by a wealth of botanical knowledge, and probably with a long history of trial and error. Indeed, the variability that we observe today in the Nyae Nyae region may reflect only a fraction of earlier technical systems, perhaps determined, in part, by different ecological niches.

As archaeologists we want to know what time depth could be associated with glues, adhesives and poisons. At present we have some archaeological evidence for the use of southern African glues, adhesives and poison, but the issue of continuity through time is not clear-cut. Part of our challenge is the absence of modern hunter-gatherers in the areas where we have archaeological evidence. Furthermore there are ecological differences between the areas where hunter-gatherers live today and where the archaeological sites are situated, so different sets of resources are applicable. The resin used as fixative at Diepkloof and Border Cave is from *Podocarpus* (a genus not present in Nyae Nyae), and the conifer resin identified on some of the Sibudu tools may also be *Podocarpus*. At Diepkloof and Border Cave, the resin may have been deliberately mixed with fragmented bone and quartz grains, and at Border Cave charcoal is a further additive, but such components can easily be contaminants, so the interpretation of compound adhesive use incorporating *Podocarpus* is not completely secure. At Sibudu, conifer resin was sometimes mixed with red ochre and animal fat. Only three Sibudu tools have successfully undergone chemical analysis, but microscopy reveals other mixtures of ingredients that appear to include ochre, plant exudates and maybe animal products. Further chemical analyses are needed on ancient tools to establish securely the technical complexity that preliminary work suggests. It is our intention, as the work develops, to conduct GC-MS in addition to other tests appropriate for the materials in hand. We shall analyse modern comparative materials as well as available archaeological residues.

While we already know that varied recipes were used in the past, we have not yet explored the possibility that different fixative pastes may have been layered on a single artefact, as has been demonstrated at Nyae Nyae. We also do not yet know for certain whether particular fixative pastes were restricted to certain artefact classes, as they are at Nyae Nyae. The Nyae Nyae fixative pastes are task-specific, for example, as mentioned earlier, there are different fixative pastes for heavy and light-duty tools and weapons. We found no compelling evidence for diverse hafting traditions in Nyae Nyae. The variability that we observed was limited to poison recipes. There seems considerable scope for idiosyncratic poison recipes and we have yet to discover whether this was also the case in the past.

The Border Cave lump of beeswax mixed with poisonous sap is undoubtedly a compound product. It is tempting to suggest that this represents combined glue and poison, a product that possibly implies technical sophistication equal to that at Nyae Nyae. Nonetheless, we are cognisant that there is little continuity between the modern and ancient practices, even after taking into account the ecological disjunctions between our ethnographic and archaeological observations. This suggests that we should exercise caution when we are tempted to assume continuity in the manufacture and uses of material culture from prehistory to the present. We are also aware that archaeological sites may not preserve some of the items that we see at Nyae Nyae today, even if they were used in the past; degradation is an issue that residue analysts constantly face. Some modern items essential for the manufacture and/or storage of products like poison mixtures, or glues, have not yet been found archaeologically. These include spatulas, hollow bones or stones used for mixing bowls, and containers or sticks for storing glue or poison. Our ethnographic observations make us realise how carefully we need to examine archaeologically recovered objects like hollow bones for residues that may suggest their use during manufacture of various substances. In addition, we need to bear in mind that some ingredients may have been brought to a site from afar. We also acknowledge that some micro-residues, like those described earlier, preserve well on stone tools, but that we are not able to detect products that have disappeared because of diagenesis or other post-depositional processes. Thus precise recipes for fixative pastes and poisons used in the past may be unrecoverable. However, what has been lost sometimes represents discontinuity in technical systems and traditions through time, for example, the Border Cave applicator stick bears poison that has no modern equivalent. Our ethnographic study has value for creating models that archaeologists can reference, but it also demonstrates that ethnography cannot be used in a simplistic way to interpret archaeological data.
